# Statin discontinuation and new antipsychotic use after an acute hospital stay vary by hospital

**DOI:** 10.1371/journal.pone.0232707

**Published:** 2020-05-08

**Authors:** Antoinette B. Coe, Brenda M. Vincent, Theodore J. Iwashyna

**Affiliations:** 1 Department of Clinical Pharmacy, College of Pharmacy and Institute for Healthcare Policy and Innovation, University of Michigan, Ann Arbor, Michigan, United States of America; 2 Center for Clinical Management Research, Veterans Affairs Ann Arbor Healthcare System, Ann Arbor, Michigan, United States of America; 3 Department of Internal Medicine and Institute for Healthcare Policy and Innovation, University of Michigan, Ann Arbor, Michigan, United States of America; Wayne State University, UNITED STATES

## Abstract

**Introduction:**

Patients are at risk for medication problems after hospital admissions, particularly those with critical illness. Medication problems include continuation of acute medications and discontinuation of chronic medications after discharge. Little is known across a national integrated health care system about the extent of these two medication problems.

**Objective:**

To examine the extent of statin medication discontinuation and new antipsychotic medication use after hospital discharge.

**Design:**

Retrospective cohort study.

**Setting:**

Veterans Affairs healthcare system.

**Participants:**

Veterans with an inpatient hospitalization from January 1, 2014-December 31, 2016, survived at least 180 days post-discharge, and received at least one medication through the VA outpatient pharmacy within one year around admission were included. Hospitalizations were grouped into: 1) direct admission to the intensive care unit (ICU) and a diagnosis of sepsis, 2) direct admission to the ICU without sepsis diagnosis, and 3) no ICU stay during the hospitalization.

**Main outcome measures:**

Statin medication discontinuation and new antipsychotic use at six months post-hospital discharge.

**Results:**

A total of 520,187 participants were included in the statin medication and 910,629 in the antipsychotic medication cohorts. Statin discontinuation ranged from 10–15% and new antipsychotic prescription fills from 2–4% across the three hospitalization groups, with highest rates in the ICU admission and sepsis diagnosis group. Statin discontinuation and new antipsychotic use after a hospitalization varied by hospital, with worse performing hospitals having 11% higher odds of discontinuing a statin (median odds ratio at hospital-level, adjusted for patient differences, aMOR: 1.11 (95% CI: 1.09, 1.13)) and 29% higher odds of new antipsychotic use (aMOR, 1.29 (95% CI: 1.24, 1.34)). Risk-adjusted hospital rates of these two medication changes were not correlated (p = 0.49).

**Conclusions:**

Systemic variation in the rates of statin medication continuation and new antipsychotic use were found.

## Introduction

Medication problems are a significant concern in the care transition after hospital admissions, particularly those including an intensive care unit (ICU) stay. Medications started in the hospital during critical illness may be inadvertently continued, such as antipsychotics or proton pump inhibitors, leading to potential harm and unnecessary costs.[1–11] Of particular concern, antipsychotics started for ICU delirium and continued after discharge are associated with adverse events such as QT_C_ prolongation, extrapyramidal symptoms, and increased risk of death in older adults.[12] Compounding the problem, chronic medications used before the ICU hospital stay may be stopped for transient reasons, but never restarted. The discontinuation of chronic medications, such as anticoagulants or statin cholesterol medications, increases risk of emergency department visits, hospitalizations, or death.[13]

With the exception of two population-based studies conducted in a single province in Canada,[13, 14] much of the work examining medication discontinuation or continuation after hospitalization or ICU stay have been conducted in one or two medical centers or for a limited amount of follow-up time.[1–11] To our knowledge, no information exists to the extent of the problem of statin discontinuation or new antipsychotic starts—used as tracer conditions for discontinuation of a chronic medication and new use of a potentially acute medication—in a national population in the United States. It is also unclear to what extent these represent a single common failure (e.g. some form of hospital-wide poor medication reconciliation) or separate error-generating processes that may require individualized interventions.

This current study sought to determine the extent of both chronic statin medication discontinuation and new antipsychotic medication use after an ICU stay compared to those hospitalized without an ICU stay in the United States Veterans Health Administration health care system. We examined if Veterans with an ICU stay and a sepsis diagnosis versus those without a sepsis diagnosis was associated with greater risk of statin discontinuation and new antipsychotic use, given the frequency of multi-organ involvement in patients with sepsis and risk for late mortality.[15–17] We hypothesized that Veterans with an ICU stay and a sepsis diagnosis would have the highest risk of statin discontinuation and new antipsychotic use, and that there would be a measurable, shared, hospital-component to the rate of these risks.

## Materials and methods

### Data source

Data from the Veterans Affairs Adult Patient Database (VAPD), containing all inpatient VA hospitalizations from January 1, 2014 through December 31, 2017 was used in this study.[18] The VAPD contains patient characteristics and information related to inpatient VA hospital admissions obtained from the VA Corporate Data Warehouse, as well as VA hospital level variables. This database was linked to VA outpatient medication data.

### Study design and cohort identification

This retrospective cohort study identified patients with an inpatient VA hospitalization between January 1, 2014 to December 31, 2016 and discharged by December 31, 2016. Inclusion criteria included survival at least 180 days post-discharge. To ensure identification of VA pharmacy users, patients had to have received at least one medication within 365 days before hospital admission and within 365 days post-discharge to be included.

Hospitalizations were categorized into three primary exposure groups: 1) direct admission to the intensive care unit (ICU) and a diagnosis of sepsis on day one, 2) direct admission to the ICU with no sepsis diagnosis on day one, and 3) no ICU stay during the entire hospitalization. Sepsis diagnosis was identified using the Centers for Disease Control and Prevention electronic health record-based definition.[18, 19]

#### Primary outcomes (1): Statin medication use discontinuation

Statin medications were identified in the outpatient VA pharmacy claims data by VA class CV350 antilipemic agents. Individual medication names in this class were then reviewed and statin medications were verified. Statin medication names included are provided in S1 Appendix.

Patients were included in the statin medication use cohort if they had a statin medication claim within 180 days prior to hospital admission. This timeframe was used to indicate use of a statin medication prior to admission. Statin discontinuation was defined as no statin dispensed within 180 days following discharge from the hospital.

#### Primary outcomes (2): Antipsychotic medication use

Antipsychotic medications were identified in the outpatient VA pharmacy claims data by VA class CN709 antipsychotics/other. Individual medication names in this class were then reviewed and antipsychotic medications were verified. Antipsychotic medication names included are also provided in S1 Appendix.

Patients were included in the antipsychotic medication use cohort if they had no antipsychotic medication claim in the 180 days prior to admission and had an antipsychotic medication claim within 180 days of discharge. This timeframe was used to indicate a new start of antipsychotic medication after a hospital admission. New antipsychotic use was defined as any antipsychotic fill within 180 days of discharge for patients.

#### Risk adjustment

Patient characteristics included were age, race, and sex. Van Walraven’s Elixhauser score was included as a comorbidity measure[20] and indicators for the top 10 most frequent admitting diagnoses (excluding sepsis) were also included. Illness severity was calculated using a variety of clinical factors including demographics, diagnoses, comorbidities and laboratory values.[21] Hospital level covariates were: hospital region (Midwest, Northeast, South, West), hospital size (small (< 200 beds), medium (200–499 beds), large (> 500 beds)), hospital complexity level (1a, 1b, 1c, 2, 3), and whether it was a teaching hospital (yes/no). Details on risk-adjustment, including analytic code, are presented in S2 Appendix.

### Statistical analysis

Descriptive statistics were used to describe the patient and hospital level characteristics in the three primary exposure groups for both the statin medication discontinuation and new antipsychotic use cohorts. Chi-square analysis separately examined differences in the proportion of new antipsychotic use and statin discontinuation between the cohorts.

Multilevel logistic models with hospitalizations nested within hospitals were used to estimate the risk- and reliability-adjusted rates of statin discontinuation and new antipsychotic use. A random intercept for hospital was used, which allows for the inclusion of hospital-level covariates and benefits from the property of shrinkage. The models were adjusted for patient (hospitalization-related) level and hospital level covariates. The least squares means for each group was calculated to determine the differences in rates of statin discontinuation and new antipsychotic use. The reliability adjusted hospital rates of statin discontinuation and new antipsychotic use was estimated using the random intercept from the multilevel model, with covariates set to the population average (details provided in S2 Appendix). The median odds ratio (MOR) was used to quantify the variation between hospitals. Correlation analysis compared statin discontinuation with new antipsychotic use at the hospital level. A post-hoc sensitivity analysis was conducted including a mental health diagnosis covariate in the multilevel model examining new antipsychotic use (details provided in S3 Appendix).

For the statin discontinuation analysis, the time since last fill was calculated as the number of days between admission and the date of the last statin medication pharmacy claim. For the antipsychotic medication use analysis, time to first fill was calculated as the number of days between the date of hospital discharge to date of first antipsychotic medication pharmacy claim. The cumulative density functions of days since last statin fill and days to first antipsychotic fill were created. SAS version 9.4 (SAS Institute Inc., Cary, NC) was used for data analysis. This study was approved by the Ann Arbor VA Institutional Review Board with a waiver of requirement for informed consent (IRB-2016-326, approved April 26, 2019).

## Results

### Statin medication discontinuation

In 2014 to 2016, there were 520,187 hospitalizations from Veterans with prior statin use that met eligibility criteria. Of these hospitalizations, 5,939 (1.1%) patients were admitted directly to the ICU and had a sepsis diagnosis, 76,303 (14.7%) were admitted directly to the ICU without a sepsis diagnosis, and 437,945 (84.2%) were never admitted to the ICU during their hospital stay. Most patients were male (96.2%), white (73.8%), and the average age was 68.4 years (SD = 10.2). **Table 1** provides patient and hospital level characteristics for the statin medication cohort. Of those who filled a statin prescription in the previous 6 months, approximately 30% had filled at least one statin prescription in the 30 days prior to their hospitalization (S4 Appendix).

**Table 1:** Patient and hospital level characteristics of the statin cohort by hospitalization type.

	ICU admission, sepsis diagnosis (n = 5,939)	ICU admission, no sepsis diagnosis (n = 76,303)	No ICU admission (n = 437,945)
Age, mean (SD)	68.5 (9.6)	67.4 (9.4)	68.5 (10.4)
Sex, N (%)			
Male	5,744 (96.7)	73,861 (96.8)	420,922 (96.1)
Female	195 (3.3)	2,442 (3.2)	17,023 (3.9)
Race, N (%)			
White	4,486 (75.5)	57,283 (75.1)	322,022 (73.5)
Black	1,073 (18.1)	14,424 (18.9)	86,896 (19.8)
Other	380 (6.4)	4,596 (6.0)	29,027 (6.6)
Illness severity, mean (SD)	0.13 (0.13)	0.05 (0.07)	0.04 (0.05)
Elixhauser Comorbidity Score, mean (SD)	9.1 (7.3)	5.9 (6.7)	5.5 (6.6)
Admission diagnosis, N (%)			
Congestive heart failure; non-hypertensive	127 (2.1)	2,940 (3.9)	31,041 (7.1)
Nonspecific chest pain	<10	1,141 (1.5)	13,679 (3.1)
Coronary atherosclerosis and other heart disease	16 (0.3)	6,447 (8.5)	14,622 (3.3)
Cardiac dysrhythmias	98 (1.7)	5,424 (7.1)	16,073 (3.7)
Alcohol-related disorders	23 (0.4)	650 (0.9)	7,403 (1.7)
Chronic obstructive pulmonary disease and bronchiectasis	186 (3.1)	1,549 (2.0)	17,439 (4.0)
Pneumonia	426 (7.2)	993 (1.3)	14,954 (3.4)
Skin and subcutaneous tissue infections	111 (1.9)	224 (0.3)	14,074 (3.2)
Osteoarthritis	<10	414 (0.5)	17,131 (3.9)
Complication of device; implant or graft	106 (1.8)	1,932 (2.5)	9,333 (2.1)
US Region, N (%)			
Midwest	1,389 (23.4)	17,141 (22.5)	100,606 (23.0)
Northeast	719 (12.1)	10,408 (13.6)	52,388 (12.0)
South	2,799 (47.1)	36,053 (47.3)	196,004 (44.8)
West	1,032 (17.4)	12,701 (16.7)	88,947 (20.3)
AHA hospital size, N (%)			
Large	1,178 (19.8)	15,430 (20.2)	93,870 (21.4)
Medium	2,302 (38.8)	32,272 (42.3)	198,760 (45.4)
Small	2,459 (41.4)	28,601 (37.5)	145,315 (33.2)
Hospital complexity, N (%)			
1a	3,058 (51.2)	41,643 (54.6)	237,008 (54.1)
1b	1,669 (28.1)	19,479 (25.5)	102,310 (23.4)
1c	817 (13.8)	9,815 (12.9)	69,877 (16.0)
2	379 (6.4)	5,225 (6.9)	18,880 (4.3)
3	16 (0.3)	141 (0.2)	9,870 (2.3)
Teaching hospital, N (%)	4,035 (67.9)	52,810 (69.2)	298,773 (68.2)
Admission year, N (%)			
2014	2,028 (34.2)	25,953 (34.0)	148,163 (33.8)
2015	1,963 (33.1)	25,296 (33.2)	144,767 (33.1)
2016	1,948 (32.8)	25,054 (32.8)	145,015 (33.1)
Hospital length of stay in days, median (IQR)	8 (5, 13)	4 (2, 6)	3 (1, 5)
ICU length of stay in days, median (IQR)	2 (1, 4)	1 (1, 3)	-
Statin discontinued, N (%)	899 (15.1)	7,611 (10.0)	50,357 (11.5)

Cells with 10 or fewer are reported as <10 to protect patient privacy. All comparisons were statistically significant at p<0.001.

The ICU admission and sepsis diagnosis group had a higher proportion of statin discontinuation following their hospital stay (15.1% never filling a single statin prescription in VA in the 180 days after discharge, despite having filled a statin prescription prior to the hospitalization) compared to those with an ICU admission without sepsis (10.0%) and the no ICU admission group (11.5%). The chi-square test for differences between the groups was significant (p<0.0001, **Table 1**).

In a fully adjusted model for patient and hospital level characteristics, the statin discontinuation rate after discharge was significantly lower in those with an ICU stay without sepsis compared to those with an ICU stay and sepsis diagnosis on admission (**Table 2**). The odds of statin discontinuation were 0.83 (95% CI: 0.77–0.90) times lower among those with an ICU stay and no sepsis diagnosis compared to those with an ICU stay and a sepsis diagnosis on admission, after adjusting for all patient and hospital level factors. No significant differences in the odds of statin discontinuation were observed in patients hospitalized without an ICU stay compared to those hospitalized with an ICU stay and sepsis diagnosis on admission.

**Table 2:** Statin discontinuation at six-months post-hospitalization.

	OR (95% CI)
ICU admission, sepsis diagnosis	1.0
ICU admission, no sepsis diagnosis	0.83 (0.77, 0.90)
Hospitalization, no ICU	1.01 (0.93, 1.08)
Age (unit = 5 years)	0.95 (0.95, 0.96)
Race	
White	1.0
Black	1.26 (1.23, 1.29)
Other	1.13 (1.09, 1.17)
Male	0.80 (0.77, 0.84)
Illness severity (unit = 0.01)	1.03 (1.02, 1.03)
Elixhauser Comorbidity Score	1.00 (1.00, 1.00)
Admission diagnosis	
Congestive heart failure; non-hypertensive	0.66 (0.64, 0.69)
Nonspecific chest pain	0.69 (0.65, 0.73)
Coronary atherosclerosis and other heart disease	0.45 (0.42, 0.48)
Cardiac dysrhythmias	0.63 (0.60, 0.67)
Alcohol-related disorders	1.66 (1.57, 1.76)
Chronic obstructive pulmonary disease and bronchiectasis	0.74 (0.70, 0.78)
Pneumonia	0.80 (0.76, 0.85)
Skin and subcutaneous tissue infections	0.95 (0.90, 1.00)
Osteoarthritis	0.71 (0.67, 0.75)
Complication of device; implant or graft	0.90 (0.84, 0.95)
Region	
West	1.0
Midwest	1.03 (0.96, 1.10)
Northeast	1.00 (0.93, 1.09)
South	1.02 (0.95, 1.09)
AHA hospital size	
Small	1.0
Medium	0.93 (0.88, 0.99)
Large	0.95 (0.88, 1.03)
Hospital complexity	
3-Least complex	1.0
2	1.03 (0.92, 1.15)
1c	1.01 (0.91, 1.12)
1b	0.99 (0.88, 1.10)
1a-Most complex	1.03 (0.93, 1.15)
Teaching hospital	0.92 (0.87, 0.98)
Admission year	
2016	1.0
2015	1.02 (1.00, 1.04)
2014	1.00 (0.98, 1.02)

For continuous variables, the odds ratios are for a 1 unit change from the mean, except for illness severity and age, which is 0.01 unit change and 5-year change from the mean, respectively.

Hospitals varied in their adjusted rates of statin discontinuation. Adjusted to the population mean of all patient factors, the 25^th^ and 75^th^ percentile (interquartile range) of hospital discontinuation rates for statins differed by 1.4 percentage points, and the 10^th^ and 90^th^ percentile differed by 2.6 percentage points. The differences in adjusted discontinuation rates between the most and least likely to discontinue quartiles of hospitals were statistically significantly different (p <0.0001). Looked at another way, of any two hospitals, the worse performing hospital had an average adjusted 11% higher odds of discontinuing a statin after a hospitalization than did the better performing hospital (median odds ratio at hospital-level, adjusted for differences in patients, 1.11 (95% CI: 1.09, 1.13)).

### New antipsychotic medication use

From 2014 to 2016, there were 910,629 hospitalizations in which the patient survived at least 180 days post-discharge, had at least one pharmacy fill in the year prior to admission and within a year post-discharge, and did not have an antipsychotic medication filled within 180 days prior to their hospitalization. Most hospitalizations did not include an ICU stay (775,630 (85.2%)). There were 10,167 (1.1%) hospitalizations directly admitted to the ICU with a sepsis diagnosis and 124,832 (13.7%) hospitalizations directly admitted to the ICU without a sepsis diagnosis. The majority of patients in the antipsychotic medication use cohort were male (94.6%), white (71.9%), and the average age was 66.3 years (SD = 12.3) (**Table 3**). Of those who would go on to fill an antipsychotic within 180 days, approximately 40% had filled an antipsychotic prescription in the 30 days after their hospitalization (S5 Appendix).

**Table 3:** Patient and hospital level characteristics of the antipsychotic cohort by hospitalization type.

	ICU admission, sepsis diagnosis (n = 10,167)	ICU admission, no sepsis diagnosis (n = 124,832)	No ICU admission (n = 775,630)
Age, mean (SD)	67.1 (10.9)	65.8 (11.1)	66.4 (12.5)
Sex, N (%)			
Male	9,801 (96.4)	119,431 (95.7)	732,195 (94.4)
Female	366 (3.6)	5,401 (4.3)	43,435 (5.6)
Race, N (%)			
White	7,361 (72.4)	91,191 (73.1)	556,433(71.7)
Black	2,092 (20.6)	25,767 (20.6)	164,473 (21.2)
Other	714 (7.0)	7,874 (6.3)	54,724 (7.1)
Illness severity, mean (SD)	0.13 (0.14)	0.05 (0.07)	0.04 (0.05)
Elixhauser Comorbidity Score, mean (SD)	9.6 (7.5)	5.9 (6.9)	5.3 (6.8)
Admission diagnosis, N (%)			
Congestive heart failure; non-hypertensive	164 (1.6)	3,947 (3.2)	41,999 (5.4)
Nonspecific chest pain	<10	1,545 (1.2)	19,866 (2.6)
Coronary atherosclerosis and other heart disease	22 (0.2)	7,935 (6.4)	18,008 (2.3)
Cardiac dysrhythmias	139 (1.4)	8,250 (6.6)	25,040 (3.2)
Alcohol-related disorders	108 (1.1)	2,345 (1.9)	27,106 (3.5)
Chronic obstructive pulmonary disease and bronchiectasis	279 (2.7)	2,512 (2.0)	27,903 (3.6)
Pneumonia	716 (7.0)	1,581 (1.3)	24,995 (3.2)
Skin and subcutaneous tissue infections	166 (1.6)	390 (0.3)	25,675 (3.3)
Osteoarthritis	<10	783 (0.6)	33,061 (4.3)
Complication of device; implant or graft	193 (1.9)	2,741 (2.2)	16,652 (2.2)
US Region, N (%)			
Midwest	2,366 (23.3)	27,735 (22.2)	175,339 (22.6)
Northeast	1,165 (11.5)	17,100 (13.7)	91,436 (11.8)
South	4,782 (47.0)	58,704 (47.0)	341,369 (44.0)
West	1,854 (18.2)	21,293 (17.1)	167,486 (21.6)
AHA hospital size, N (%)			
Large	2,095 (20.6)	25,078 (20.1)	165,308 (21.3)
Medium	3,977 (39.1)	53,389 (42.8)	356,376 (46.0)
Small	4,095 (40.3)	46,365 (37.1)	253,946 (32.7)
Hospital complexity, N (%)			
1a	5,381 (52.9)	68,203 (54.6)	423,316 (54.6)
1b	2,742 (27.0)	31,504 (25.2)	179,080 (23.1)
1c	1,403 (13.8)	16,181 (13.0)	121,228 (15.6)
2	609 (6.0)	8,678 (7.0)	34,058 (4.4)
3	32 (0.3)	266 (0.2)	17,948 (2.3)
Teaching hospital, N (%)	6,967 (68.5)	85,553 (68.5)	525,893 (67.8)
Admission year, N (%)			
2014	3,464 (34.1)	42,734 (34.2)	264,723 (34.1)
2015	3,354 (33.0)	41,409 (33.2)	256,473 (33.1)
2016	3,349 (32.9)	40,689 (32.6)	254,434 (32.8)
Hospital length of stay in days, median (IQR)	8 (5, 13)	4 (2, 7)	3 (1, 5)
ICU length of stay in days, median (IQR)	2 (1, 4)	1 (1, 3)	-
Antipsychotic new fill	361 (3.6)	2,917 (2.3)	21,703 (2.8)

All comparisons were statistically significant at p<0.001.

A higher proportion of new antipsychotic prescription use among those with no outpatient antipsychotic prescriptions in the 180 days prior to hospitalization was observed in the ICU admission and sepsis diagnosis group following their hospital stay (3.6%) compared to those with an ICU admission without sepsis (2.3%) and the no ICU admission group (2.8%). A chi-square test for differences between the groups was significant (p<0.0001, **Table 3**).

In a model adjusting for patient and hospital level characteristics, new antipsychotic fills within 180 days after discharge was lower in those with an ICU stay without a sepsis diagnosis compared to those without an ICU stay and sepsis diagnosis on admission (**Table 4**). Those with an ICU stay without a sepsis diagnosis on admission had 0.76 times lower odds of having a new antipsychotic filled (95% CI: 0.68–0.85) compared to those admitted to the ICU with a sepsis diagnosis after adjusting for all patient and hospital level factors. No significant differences in the odds of a new antipsychotic medication fill were observed in patients hospitalized without an ICU stay compared to those hospitalized with an ICU stay and sepsis diagnosis on admission.

**Table 4:** New antipsychotic use in the year following hospitalization.

	OR (95% CI)
ICU admission, sepsis diagnosis	1.0
ICU admission, no sepsis diagnosis	0.76 (0.68, 0.85)
Hospitalization, no ICU	0.91 (0.81, 1.01)
Age (unit = 5 years)	0.91 (0.90, 0.91)
Race	
White	1.0
Black	1.07 (1.04, 1.11)
Other	0.98 (0.93, 1.03)
Male	0.88 (0.82, 0.91)
Illness severity (unit = 0.01)	1.03 (1.03, 1.03)
Elixhauser-Van Walraven Score	0.97 (0.96, 0.97)
Admission Diagnosis	
Congestive heart failure; non-hypertensive	0.69 (0.64, 0.75)
Nonspecific chest pain	1.00 (0.92, 1.08)
Coronary atherosclerosis and other heart disease	0.57 (0.52, 0.63)
Cardiac dysrhythmias	0.51 (0.46, 0.56)
Alcohol-related disorders	2.75 (2.63, 2.88)
Chronic obstructive pulmonary disease and bronchiectasis	0.77 (0.71, 0.84)
Pneumonia	0.72 (0.66, 0.78)
Skin and subcutaneous tissue infections	0.70 (0.64, 0.76)
Osteoarthritis	0.28 (0.25, 0.32)
Complication of device; implant or graft	0.66 (0.59, 0.73)
Region	
West	1.0
Midwest	1.01 (0.85, 1.19)
Northeast	1.13 (0.94, 1.37)
South	1.15 (0.99, 1.34)
AHA hospital size	
Small	1.0
Medium	1.01 (0.88, 1.15)
Large	0.86 (0.72, 1.04)
Hospital complexity	
3-Least complex	1.0
2	0.76 (0.60, 0.96)
1c	0.86 (0.69, 1.06)
1b	0.66 (0.52, 0.83)
1a-Most complex	0.77 (0.61, 0.96)
Teaching hospital	0.99 (0.85, 1.15)
Admission year	
2016	1.0
2015	1.04 (1.00, 1.07)
2014	1.03 (0.99, 1.06)

For continuous variables, the odds ratios are for a 1 unit change from the mean, except for illness severity and age, which is 0.01 unit change and 5-year change from the mean, respectively.

Hospitals varied in their adjusted rates of new antipsychotic use. Adjusted to the population mean individual-levels of all patient factors, the 25^th^ and 75^th^ percentile (interquartile range) of hospital new antipsychotic use differed by 0.8 percentage points, and the 10^th^ and 90^th^ percentile different by 1.6 percentage points. The differences in adjusted continuation rates between the most and least likely to continue quartiles of hospital were statistically significantly different (p <0.0001). Looked at another way, of any two hospitals, the worse performing hospital had an adjusted 29% higher odds of new antipsychotic use after a hospitalization than did the better performing hospital (median odds ratio at hospital-level, adjusted for differences in patients, 1.29 (95% CI: 1.24, 1.34)). The estimated median odds ratio did not change in a post-hoc sensitivity analysis that adjusted for patient-level mental health diagnoses (S3 Appendix).

**Fig 1:**
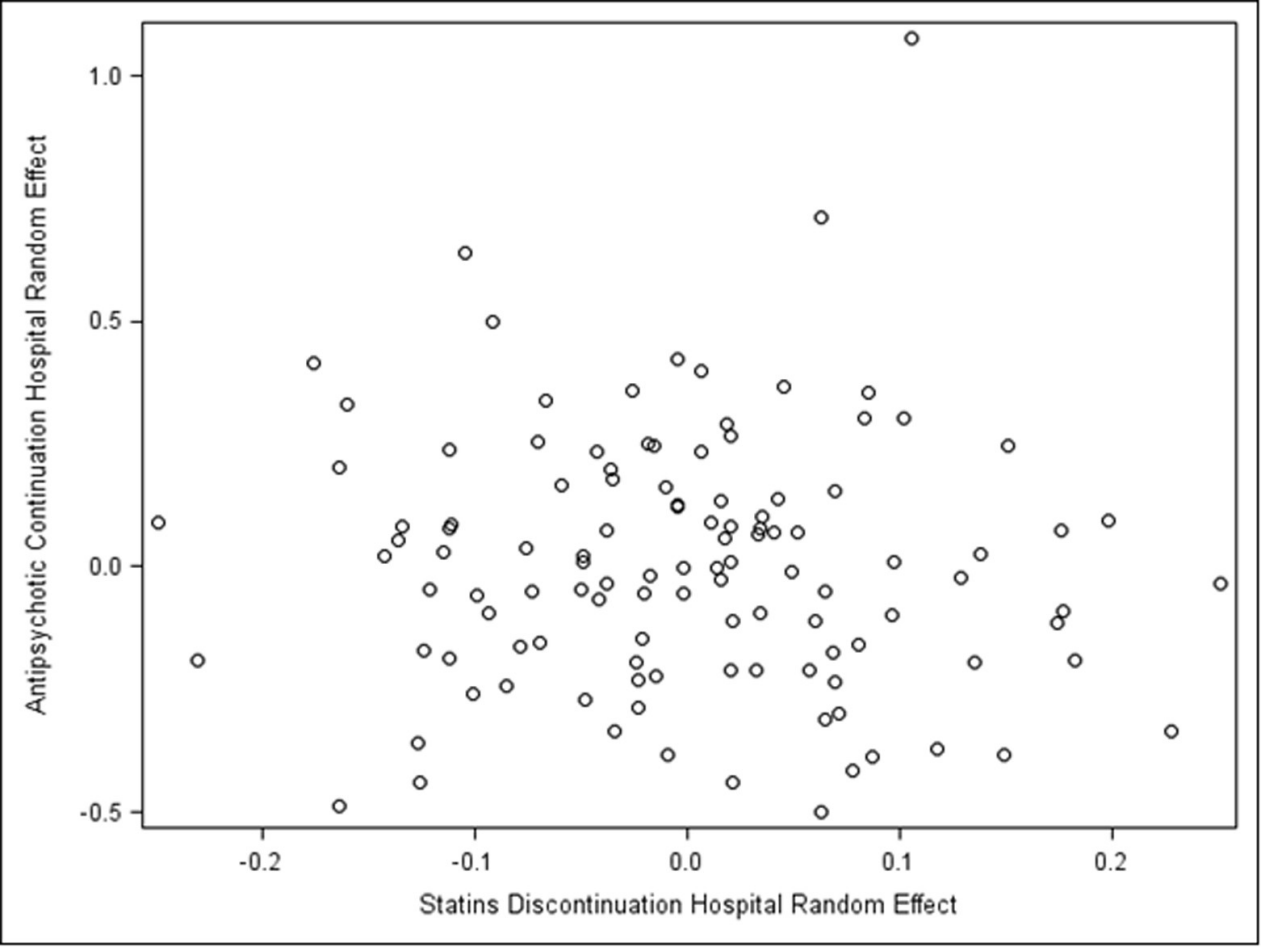
Correlation between statin discontinuation and antipsychotic continuation at VA hospitals. Adjusted for all patient-level factors and on the log-odds scale.

In 114 VA hospitals, the correlation between adjusted statin discontinuation and adjusted new antipsychotic use hospital effects was -0.06 (p = 0.49, **Fig 1**).

### Sensitivity analyses

We tested other possible definitions of lookback periods prior to hospitalization (30 or 365 days) as well as surveillance periods after hospitalization (30 or 365 days). While there was expected variation in the precise odds ratios, the general pattern of higher rates in ICU-Sepsis, and of hospital-level variation, was present for both medications across each analysis. In some cases, there was modest correlation (up to 0.11) between adjusted statin discontinuation and adjusted new antipsychotic use at the hospital effects.

## Discussion

Among patients discharged from a VA hospital, we found up to 15.1% of previously statin-using Veterans did not have a statin medication filled and up to 3.6% of previously antipsychotic-naïve Veterans had a new antipsychotic medication filled within six months post-discharge. Additionally, the rates of statin discontinuation and new antipsychotic starts were highest in those with sepsis and an ICU stay. These two medication changes are of particular importance due to their association with increased emergency department visits, hospitalizations, cardiovascular and metabolic side effects, and mortality—but may also serve as markers of broader potential dysfunction.[13, 22, 23]

This study supports the need for new approaches to medication reconciliation and review of medications in acute illness survivors, especially after more severe or critical illness. Medication problems, including both actual and potentially inappropriate medication use, are common after hospital discharge for acute illness.[5, 6] However, due to hospital survivors’ complex and dynamic physiological state—particularly prominent in those surviving critical illness and/or sepsis—restarting chronic medications, such as statins or blood pressure medications, at the time of discharge may not yet be appropriate. This may account for a higher proportion of statin discontinuation in this study. Likewise, the cognitive impairment and impact on mental health seen in hospital survivors,[24] and especially post-ICU survivors,[25] such as new onset depression, post-traumatic stress disorder, or anxiety, may necessitate outpatient follow-up for evaluation of newly added psychotropic medications for potential deprescribing and tapering.[10, 26, 27] The appropriate time to address certain medication problems and fully optimize medication use may be after the patient’s transition to home, but this is not well studied. Interprofessional transition and post-ICU recovery clinics may be the optimal setting to address the health, medication, and social needs of post-ICU survivors.[10, 28, 29] In one post-ICU recovery clinic, a clinical pharmacist intervened on a median of four medication problems per patient, including stopping a medication at 39% of patient visits indicating the benefit of a medication review in this population.[10] For Veteran post-ICU survivors, follow-up within the primary care-mental health integration (PCMHI) program may be an option to address their mental health medication changes post-discharge.[30]

Our identified rates of statin medication discontinuation and new antipsychotic use are consistent with those seen in large population-based cohort studies [13, 14] and have several potential mechanisms. First, statins may have several reasons for discontinuation during hospitalization, such as infection or appropriate discontinuation near end of life.[31, 32] Likewise, there may be an acute need for an antipsychotic during hospitalization, such as delirium.[2, 3, 9, 11, 12, 33] The indications for use of both medications are unclear from the outpatient pharmacy medication fill records used in this study. However, our work is the first to show hospital-level variation in the discontinuation of statin medication use and new antipsychotic medication use after discharge, even after severity of illness adjustment and sensitivity analysis. Our findings of variations across hospitals indicate that there is more at play than the individual patient factor-related proposed mechanisms. Other studies have shown that hospital variation exists in the VA, such as in hospital cardiac arrest incidence and outcomes [34] and practices around non-formulary medication use.[35] Additional research is needed to characterize individual hospital practices, such as systematic medication reconciliation practices around each care transition, at discharge, and at first primary care visit; variation in health professional staffing; or local quality improvement initiatives, which may be influencing these medication use outcome differences across the VA system.

Our findings are consistent with previous individual-level literature indicating that statin medications, along with other chronic medications, were discontinued after an ICU or hospital admission and new antipsychotic use occurs after discharge.[10, 13, 14] We found similar rates of statin discontinuation as other work (10–15% vs. 14%[13]); however, our study included a broader age range and examined medication claims over a longer post-discharge time period (6 months vs. 3 months). Other studies examining rates of discharge on antipsychotics started in the ICU found approximately 21–33% of patients were discharged home on an antipsychotic; whereas our results indicated 2.3–3.6% of patients had a new antipsychotic medication filled in 180 days after discharge. [3, 33, 36] This is further evidence of system-level differences in practice that warrant exploration to identify positive deviants and disseminate their practices.

Strengths of this study include the use of a large cohort of Veterans to examine rates of statin medication discontinuation and new antipsychotic use after critical illness in United States’ largest integrated health system. We also adjusted for patient-level severity and hospital-level variables allowing for comparison across individual VA hospitals. The use of outpatient pharmacy claims is another strength as it allowed us to see patterns of medication fills over several months post-discharge.

Our study has several limitations. First, we used VA outpatient pharmacy claims to identify statin and antipsychotic medication use which may underestimate out-of-system medication use and are limited by information in this data. But, it is likely that Veterans had their medications filled through the VA system due to potentially lower costs than an outside pharmacy, and it is not clear why such patterns would be differential before versus after a hospitalization. We also do not have information about medication use during the hospital admission. Second, we used administrative data which may under or overestimate patients identified with sepsis. There is also the potential that patients who developed sepsis during their hospital stay were not identified since we identified sepsis diagnosis on day one. However, we used similar methods to other studies.[18, 19] Our data and those of others indicate that less than 25% of ICU sepsis is not present on ICU admission. As such, while we acknowledge there is some potential for misclassification, its magnitude is modest—and would tend to attenuate our results to the null, but would not change our substantive interpretations at either the patient or, more importantly, the hospital level. Third, we were able to identify variation between hospitals in the VA health system but were unable to attribute that variation to individual provider prescribing patterns or behaviors within the hospital.

## Conclusions

Systemic variation in the rates of statin medication continuation and new antipsychotic use were found. Patients with an ICU admission and sepsis diagnosis were at highest risk for statin medication discontinuation and new antipsychotic medication fills across a large, integrated health care system. Systemic solutions (e.g. medication reviews for appropriateness and optimization of therapy) after hospital discharge may be warranted.

## Supporting information

S1 Appendix List of medications included in statin and antipsychotic medication cohorts. (DOCX)

S2 Appendix Details on risk adjustment and reliability adjustment. (DOCX)

S3 Appendix Post-hoc sensitivity analysis for new antipsychotic use cohort. (DOCX)

S4 Appendix Cumulative density function of statin fills prior to hospitalization. (DOCX)

S5 Appendix Cumulative density function of antipsychotic fills after hospitalization. (DOCX)
