# Supplementary material for: Statin discontinuation and new antipsychotic use after an acute hospital stay vary by hospital
Source: PLoS One. 2020 May 8;15(5):e0232707. doi: 10.1371/journal.pone.0232707 (PMC7209203; doi:10.1371/journal.pone.0232707)
Supplement: S1 Appendix — (DOCX) [file pone.0232707.s001.docx]

**S1 Appendix 1. List of medications included in statin and antipsychotic medication cohorts**

| **Statin Medication Name** | **Antipsychotic Medication Name^a^** |
| --- | --- |
| Atorvastatin | Aripiprazole |
| Lovastatin | Asenapine |
| Pravastatin | Brexpiprazole |
| Rosuvastatin | Cariprazine |
| Simvastatin | Clozapine |
| Ezetimibe/Simvastatin | Haloperidol |
| Lovastatin/Niacin | Iloperidone |
|  | Loxapine |
|  | Lurasidone |
|  | Molindone |
|  | Olanzapine |
|  | Paliperidone |
|  | Pimavanserin |
|  | Quetiapine |
|  | Risperidone |
|  | Ziprasidone |

^a^ Names of antipsychotic medications in CN709 antipsychotics/other
