# Supplementary material for: Statin discontinuation and new antipsychotic use after an acute hospital stay vary by hospital
Source: PLoS One. 2020 May 8;15(5):e0232707. doi: 10.1371/journal.pone.0232707 (PMC7209203; doi:10.1371/journal.pone.0232707)
Supplement: S2 Appendix — (DOCX) [file pone.0232707.s002.docx]

**S2 Appendix 2. Details on risk adjustment and reliability adjustment**

We were interested in the hospital variation in risk- and reliability-adjusted rates of statin discontinuation (and without loss of generality, new antipsychotic use) after discharge. We used a multilevel logistic model with hospitalizations nested within hospitals. As we sought to quantify hospital variation on rates of statin discontinuation, we needed to adjust for potential differences in hospitalization- and hospital-level covariates across hospitals.

To do this, we first computed a severity of illness measure for each hospitalization by predicting 30-day mortality using a variety of clinical factors (see section 1). This risk score, along with other hospitalization- and hospital-level covariates, was then included in the subsequent multilevel model to account for potential differences in case-mix across hospitals (see section 2). Using the results of the multilevel model, we computed a measure that characterized hospital-level variation, the median odds ratio (MOR). We also created a caterpillar plot that displays the risk- and reliability-adjusted rates of statin discontinuation (and 95% confidence intervals) of each hospital. In this document, we illustrate our method and include the necessary SAS code.

**Section 1: Risk adjustment**

Our goal was to adjust for hospitalization- and hospital-level covariates to account for differences in case-mix. Using the dataset that contains all hospitalizations, we used a severity of illness measure for each hospitalization at the time of admission. The severity of illness measure is defined as the predicted probability of 30-day mortality (from admission) using a logistic model that adjusts for hospitalization-level risk factors at the time of admission. Our model for severity of illness includes the following hospitalization-level covariates (see Table 1A for more details):

**Demographics**: Age, sex, race

**Diagnoses**: all granular diagnosis groups and the top 20 most frequent specific diagnoses. The granular diagnosis groups are the level 1 diagnoses from Healthcare Cost and Utilization (HCUP)^1^ multilevel Clinical Classification Software (CCS) and the specific diagnoses groups are the single-level CCS diagnosis groups.

**Comorbidities**: Indicators for 30 comorbidities (included in Elixhauser^2,3^)

**Laboratory** **values**: Acute Physiology and Chronic Health Evaluation (APACHE) III scores^4^ for labs drawn on the first calendar day of admission. These labs include albumin, bilirubin, blood urea nitrogen, creatinine, glucose, hematocrit, partial pressure of oxygen (PaO_2_), partial pressure of carbon dioxide (PaCO_2_) and pH, sodium, and white blood cell. For hospitalizations with multiple labs drawn on the first day, we used the highest value for all labs except for pH, in which we use the lowest. For hospitalizations that did not have a lab drawn in the first calendar day of admission, we assumed that that lab was “normal” and had an APACHE III score of 0.^4,5^

We used the APACHE III scores for labs instead of the raw lab values to consider the nonlinear relationships that some labs have with 30-day mortality. The overall c-statistic of our illness severity measure is 0.849. The top 20 single-level diagnoses accounted for 49.8% of hospitalizations.

We used the severity of illness measure in our models along with the individual hospitalization and hospital covariates. Hospital covariates included: region, hospital complexity, hospital, and an indicator for teaching hospital (Table 1B).

The SAS code used to generate illness severity (variable name: **va_risk_score**):

**proc** **logistic** data=vapd;

class male black white

chf_nonhp sepsis alcohol dysrhythmia pneumonia copd coron_athero osteoarthros skin_infection chestpain complic_devi uti diabmel_w_cm complic_proc acute_ren_fail backproblem acute_mi acute_cerebrovascular gi_hemorrhag adlt_resp_fl infect_parasitic_dis neoplasms endocrine blood_dis mental_illness nervous_dis circulatory respiratory digestive genitourinary pregnancy skin musculoskeletal congenital_anomalies perinatal injury_poisoning illdefined_conditions unclassified;

model mort30(event='1’) = age male black white

/*lab APACHE III scores*/

albval_sc bili_sc bun_sc creat_sc glucose_sc hct_sc na_sc pao2_sc ph_sc wbc_sc

/*comorbidities indicators*/

elx_grp_1 elx_grp_2 elx_grp_3 elx_grp_4 elx_grp_5 elx_grp_6 elx_grp_7 elx_grp_8 elx_grp_9 elx_grp_10 elx_grp_11 elx_grp_12 elx_grp_13 elx_grp_14 elx_grp_15 elx_grp_16 elx_grp_17 elx_grp_18 elx_grp_19 elx_grp_20 elx_grp_21 elx_grp_22 elx_grp_23 elx_grp_24 elx_grp_25 elx_grp_26 elx_grp_27 elx_grp_28 elx_grp_29 elx_grp_30

/*top 20 diagnosis groups of single level CCS*/

chf_nonhp sepsis alcohol dysrhythmia pneumonia copd coron_athero osteoarthros skin_infection chestpain complic_devi uti diabmel_w_cm complic_proc acute_ren_fail backproblem acute_mi acute_cerebrovascular gi_hemorrhag adlt_resp_fl

/*level 1 of multi-level CCS*/

infect_parasitic_dis neoplasms endocrine blood_dis mental_illness nervous_dis circulatory respiratory digestive genitourinary skin musculoskeletal congenital_anomalies perinatal injury_poisoning illdefined_conditions unclassified;

output out=vapd2 predicted=va_risk_score;

**run**;

**Section 2: Reliability adjustment**

We used reliability adjustment to avoid overestimating the statin discontinuation rates at hospitals with low case volume.^6^ To do this, we used a multilevel model with a random intercept for hospital. The three main advantages^7^ of using a random effects model (as opposed to including hospital as a fixed effect) are 1) to reduce the number of parameters estimated, 2) to adjust for hospital level covariates, and 3) to benefit from the property of shrinkage. In a random effects model, rather than estimate a parameter for each hospital, we simply estimate the variation in hospital effects. To adjust for hospital-level covariates, a random effects model must be used because using hospital as a fixed effect would result in collinearity with hospital-level covariates. Finally, hospital effects are assumed to arise from the same distribution, thus the effects at smaller hospitals can be informed by the entire population. Using shrinkage, hospital-specific estimates shrink toward the overall mean, and we are provided with an estimate for the underlying rate at a hospital that lies between the estimate made using only the data and the overall event rate pooled across all hospitals.

**Hospital-level variation after risk and reliability adjustment**

We quantify hospital-level variation in the outcome using the median odds ratios^8^ (MOR) calculated from the multilevel logistic regression models. The MOR can be interpreted as the odds that two patients with identical patient-level covariates from separate, randomly chosen hospitals will experience the outcome of interest. The MOR is a function of the hospital-level variance ($\mathrm{var}_{2})$ using the following formula:

$$MOR=\exp\left( 0.6745\sqrt{2(\mathrm{var}_{2}} \right).$$

Additionally, we were interested in adjusted rates of the outcome for each hospital. To do this, we set the patient-level covariates to the population means. These adjusted event rates are thus interpretable over a balanced population.

**The procedure used to obtain the adjusted event rates for each hospital (using the same hospitalization dataset)**:

1. Calculate the population means of all patient-level covariates. To do this, each categorical variable must be coded as indicator variables.
2. Fit an adjusted multilevel logistic model with hospitalization-level (including severity of illness) and hospital-level covariates, and a random intercept for hospital.
3. Estimate the log-odds of statin discontinuation at each hospital with covariates set to the population means.
4. Convert the log-odds (and 95% CI) to the probability scale by taking the inverse log.
5. Calculate the MOR and the 95% CI for MOR (by using the confidence limits of the hospital variance in the MOR equation).
6. Sort the dataset from smallest event rate to largest and create a hospital ID based on this ranking.
7. Create a caterpillar plot of the adjusted event rates and 95% CI.

**SAS code**

/*Population means*/

**proc** **means** data=all_cohorts_statin mean stackods;

var cohort1 cohort2 cohort3 VA_risk_score admityear14 admityear15 admityear16 age black white male elixhauser chf_nonhp alcohol osteoarthros dysrhythmia copd pneumonia coron_athero skin_infection chestpain complic_devi;

ods output summary=means (drop=label);

**run**;

**proc** **print** data=means noobs; **run**;

/*Create a macro for the estimate statement. Estimate the adjusted event rate (probability scale due to the ilink command) of each hospital with all individual-level covariates set to population means*/

%macro *doests*;

%do hosp=1 %to 114;

estimate "&hosp." int 1 cohort1 0.011417 cohort2 0.146684 cohort3 0.841899 VA_risk_score 0.041236 admityear14 0.338617 admityear15 0.330700 admityear16 0.330683 age 68.350857 black 0.196839 white 0.737794 male 0.962167 elixhauser_VanWalraven 5.625567 chf_nonhp 0.065569 alcohol 0.015525 osteoarthros 0.033730 dysrhythmia 0.041514 copd 0.036860 pneumonia 0.031475 coron_athero 0.040534 skin_infection 0.027700 chestpain 0.028494 complic_devi 0.021859 |intercept 1/subject %do k=1 %to %eval(&hosp.-1); 0 %end; 1 ilink e cl;

%end;

%mend;

ods output solutionr=randomeffect CovParms=cov estimates=estimates_meanpop;

**proc glimmix** data=all_cohorts_statin method=laplace;

class sta3n region hospital_complexity aha_hosp_size;

model statin_discontinued(event='1')=cohort1 cohort2 cohort3 VA_risk_score admityear14 admityear15 admityear16 age black white male elixhauser chf_nonhp alcohol osteoarthros dysrhythmia copd pneumonia coron_athero skin_infection chestpain complic_devi region aha_hosp_size hospital_complexity teaching / link=logit dist=binary solution cl ddfm=bw;

random intercept/sub=sta3n solution cl;

%*doests*; /*Call the macro that generates the estimate statements*/

**run**;

/*Sort by event rate*/

**proc** **sort** data=estimates_meanpop; by mu; **run**;

/*Create a hospital ID that is sorted from lowest to highest event rate*/

**data** estimates_meanpop;

set estimates_meanpop;

hosp=_n_;

**run**;

/*Calculate the Median Odds Ratio*/

**data** cov;

set cov;

lowerci=estimate-**1.96***stderr;

upperci=estimate+**1.96***stderr;

mor=exp(**0.6745***sqrt(**2***estimate));

mor_l=exp(**0.6745***sqrt(**2***lowerci));

mor_u=exp(**0.6745***sqrt(**2***upperci));

**run**;

/*Caterpillar plot of hospital event rates (adjusted for case mix, i.e. after reliability adjustment). Each dot and corresponding vertical line represent one hospital*/

**proc** **sgplot** data=estimates_meanpop;

scatter x=hosp y=mu/legendlabel="Adjusted Event Rate" jitter;

highlow x=hosp low=lowermu high=uppermu/legendlabel="95% CI";

yaxis label="Event Rate" values=(**0** to **1** by **0.1**);

xaxis display=none;

**run**;

**Caterpillar Plot**

**
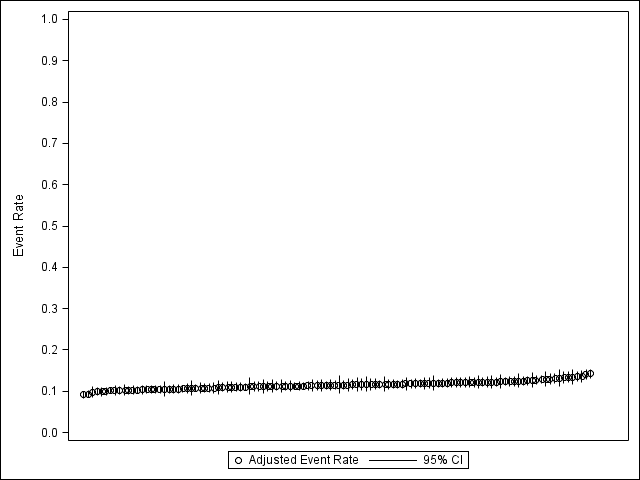
**

| Table 1A: Patient-level variables included in the risk-adjustment and reliability-adjustment models | | |
| --- | --- | --- |
| Severity of illness | VA risk score | Predicted probability of 30-day mortality from a logistic regression model that includes demographics, diagnoses, laboratory values and comorbidities at time of admission. |
| Demographics | age  sex  race^†^ | Age in years at admission  Indicator for male  Indicators for black and white |
| Diagnoses | Indicators for all level 1 diagnoses from multi-level CCS;  Indicators for the top 20 most frequent single-level CCS diagnoses | The top 20 most frequent single-level CCS diagnoses are re-generated each year.  There are 18 level 1 multilevel CCS variables which group the diagnoses into broad categories. See Table 3 for the full list. |
| Laboratory values | APACHE III scores for labs drawn on the first calendar day of admission. | albumin, bilirubin, blood urea nitrogen, creatinine, glucose, hematocrit, partial pressure of oxygen (PaO_2_), partial pressure of carbon dioxide (PaCO_2_) and pH, sodium, and white blood cell. See Table 2 for the list. |
| Comorbidities | Indicators for the 30 comorbidities included in Elixhauser^2,3^ | Congestive Heart Failure, Cardiac Arrhythmia, Valvular Disease, Pulmonary Circulation Disorders, Peripheral Vascular Disorders, Hypertension^‡^, Paralysis, Other Neurological Disorders, Chronic Pulmonary Disease, Diabetes Uncomplicated, Diabetes Complicated, Hypothyroidism, Renal Failure, Liver Disease, Peptic Ulcer Disease excluding bleeding, AIDS/HIV, Lymphoma, Metastatic Cancer, Solid Tumor without Metastasis, Rheumatoid Arthritis/collagen, Coagulopathy, Obesity, Weight Loss, Fluid and Electrolyte Disorders, Blood Loss, Anemia, Deficiency Anemia, Alcohol Abuse, Drug Abuse, Psychoses, Depression |

*Note*: We use define hospital using their STA3N (the parent hospital) for this analysis. In future work, we will use STA6A (the child hospital) to define hospital.

^†^ In future analyses, we will use an expanded definition of race with White as the reference. We will also include an indicator for Hispanic ethnicity.

^‡^ Hypertension is combined into a single comorbidity as is done in Van Walraven et al.

| Table 1B: Facility-level variables included in the risk-adjustment/reliability-adjustment models | | |
| --- | --- | --- |
| Hospital Complexity | Hospital complexity grouping from the Facility Complexity Model (1a, 1b, 1c, 2, or 3), with 1a being the most complex and level 3 being the least complex | Provided by the VHA Office of Productivity, Efficiency and Staff (OPES). The VHA Facility Complexity Model was first adopted for use in 1989. The facility groups are used for various peer grouping purposes, such as operational reporting, performance measurement, and research studies. The model is reviewed and updated with current data every 3 years (last updated: FY2017). More details are provided on the VA intranet at <http://opes.vssc.med.va.gov/FacilityComplexityLevels/Pages/default.aspx> |
| Region | Uses the U.S. Census Bureau’s classification of the geographical region for the VA facility | Midwest: Illinois, Indiana, Iowa, Kansas, Michigan, Minnesota, Missouri, North Dakota, Nebraska, Ohio, South Dakota, Wisconsin  Northeast: Connecticut, Massachusetts, Maine, New Hampshire, New Jersey, New York, Pennsylvania, Rhode Island, Vermont  South: Alabama, Arkansas, District of Columbia, Delaware, Florida, Georgia, Kentucky, Louisiana, Maryland, Mississippi, North Carolina, Oklahoma, Puerto Rico, South Carolina, Tennessee, Texas, Virginia, West Virginia  West: Alaska, Arizona, California, Colorado, Hawaii, Idaho, Montana, New Mexico, Nevada, Oregon, Utah, Washington, Wyoming  Regions available at:  United States Census Bureau, Geography Division. ["Census Regions and Divisions of the United States"](http://www2.census.gov/geo/pdfs/maps-data/maps/reference/us_regdiv.pdf) (PDF). Retrieved 4/11/19. |
| Hospital size^*^ | Defined the size of the hospital based on the number of beds in use at the facility categorized into small, medium or large. | Small <200 beds  Medium 200-499 beds  Large >500 beds |
| Teaching hospital | Indicator for teaching hospital | We use Association of American Medical Colleges (AAMC) Council of Teaching Hospitals and Health Systems (COTH) membership to identify teaching hospitals. We used a list from 2007 and January 2019. We considered all facilities that were COTH members in 2007 or 2019 as teaching hospitals regardless of their 2019 COTH status. We do this to capture the facilities that are teaching hospitals but may not have paid their dues in 2019. The current list of COTH members is available at <https://members.aamc.org/eweb/DynamicPage.aspx?webcode=AAMCOrgSearchResult&orgtype=Hospital%2FHealth%20System> |

*Note*: In future projects, we will use the annual number of single-site hospitalizations to classify facility size (Small <2000, Medium (2,000-5,000) and Large (>5,000)

| Table 2: APACHE III scores for laboratory values | | | |
| --- | --- | --- | --- |
|  | Units | Laboratory values | APACHE III Score |
| Albumin | g/l | <2.0  2-2.4  2.5-4.4  >4.4 | 11  6  0  4 |
| Bilirubin | mg/dl | <2.0  2.0-2.9  3.0-4.9  5.0-7.9  >7.9 | 0  5  6  8  16 |
| Blood urea nitrogen | mg/dl | <17.0  17-19  20-39  40-79  >79 | 0  2  7  11  12 |
| Creatinine | mg/dl | <0.4  0.4-1.50  1.51-1.94  >1.94 | 3  0  4  7 |
| Glucose | mg/dl | <40  40-59  60-199  200-349  >350 | 19  18  0  3  5 |
| Hematocrit | % | <41 or >49  41-49 | 3  0 |
| PaO_2_ | mmHg | <50  50-69  70-79  >79 | 15  5  2  0 |
| PaCO_2_ and pH | mmHg; pH | <50; <7.2  <30; 7.20-<7.35  <30; 7.35-<7.50  30-40; 7.20-<7.30  30-45; 7.30-<7.45  30-35; 7.45-<7.50  35-45; 7.45-<7.50  <40; 7.50-<7.60  25-40; >7.60  <25; >7.65  40-50; 7.20-<7.30  >50; 7.2-<7.3  >45; 7.30-<7.45  >50; <7.2  >45; 7.45-<7.50  >40; >7.50 | 12  9  5  6  0  0  2  3  3  0  3  2  1  4  12  12 |
| Sodium | mmol/l | <120  120-134  135-154  >154 | 3  2  0  4 |
| White blood cell count | X10^3^ cu mm | <1.0  1.0-2.9  3.0-19.9  20.0-24.9  >24.9 | 19  5  0  1  5 |

| **Table 3: Diagnoses included in the risk adjustment model** | |
| --- | --- |
| Level 1 of the multilevel CCS diagnoses | Top 20 single-level CCS diagnosis |
| 1. Infectious and parasitic diseases 2. Neoplasms 3. Endocrine; nutritional; and metabolic diseases and immunity disorders 4. Anemia 5. Mental illness 6. Diseases of the nervous system and sense organs 7. Diseases of the circulatory system 8. Diseases of the respiratory system 9. Diseases of the digestive system 10. Diseases of the genitourinary system 11. Complications of pregnancy; childbirth; and the puerperium 12. Diseases of the skin and subcutaneous tissue 13. Diseases of the musculoskeletal system and connective tissue 14. Congenital anomalies 15. Certain conditions originating in the perinatal period 16. Injury and poisoning 17. Symptoms; signs; and ill-defined conditions and factors influencing health status 18. Residual codes; unclassified; all E codes [259. and 260.] | 1. Congestive heart failure; non-hypertensive 2. Nonspecific chest pain 3. Coronary atherosclerosis and other heart disease 4. Cardiac dysrhythmias 5. Alcohol-related disorders 6. Septicemia (except in labor) 7. Chronic obstructive pulmonary disease and bronchiectasis 8. Pneumonia 9. Skin and subcutaneous tissue infections 10. Osteoarthritis 11. Complication of device; implant or graft 12. Complications of surgical procedures or medical care 13. Diabetes mellitus with complications 14. Respiratory failure; insufficiency; arrest (adult) 15. Urinary tract infections 16. Acute and unspecified renal failure 17. Spondylosis; intervertebral disc disorders; other back problems 18. Acute myocardial infarction 19. Fluid and electrolyte disorders 20. Gastrointestinal hemorrhage |

**References:**

1. HCUP. Healthcare Cost and Utilization Project (HCUP). Agency for Healthcare Research and Quality. [www.hcup-us.ahrq.gov/home.jsp](file:///C:\Users\VHAANNVinceB\AppData\Local\Microsoft\Windows\Temporary%20Internet%20Files\Content.Outlook\05EG4RZH\www.hcup-us.ahrq.gov\home.jsp). Published March 2019. Accessed 3/25/2019.

2. Elixhauser A, Steiner C, Harris DR, Coffey RM. Comorbidity Measures for Use with Administrative Data. *Med Care.* 1998;36(1):8-27.

3. van Walraven C, Austin PC, Jennings A, Quan H, Forster AJ. A modification of the Elixhauser comorbidity measures into a point system for hospital death using administrative data. *Med Care.* 2009;47(6):626-633.

4. Render ML, Welsh DE, Kollef M, et al. Automated computerized intensive care unit severity of illness measure in the Department of Veterans Affairs: preliminary results. SISVistA Investigators. Scrutiny of ICU Severity Veterans Health Sysyems Technology Architecture. *Crit Care Med.* 2000;28(10):3540-3546.

5. Harrell FE, Jr., Lee KL, Mark DB. Multivariable prognostic models: issues in developing models, evaluating assumptions and adequacy, and measuring and reducing errors. *Stat Med.* 1996;15(4):361-387.

6. Hayward RA, Heisler M, Adams J, Dudley RA, Hofer TP. Overestimating outcome rates: statistical estimation when reliability is suboptimal. *Health Serv Res.* 2007;42(4):1718-1738.

7. MacKenzie TA, Grunkemeier GL, Grunwald GK, et al. A Primer on Using Shrinkage to Compare In-Hospital Mortality Between Centers. *Ann Thorac Surg.* 2015;99(3):757-761.

8. Merlo J, Chaix B, Yang M, Lynch J, Rastam L. A brief conceptual tutorial of multilevel analysis in social epidemiology: linking the statistical concept of clustering to the idea of contextual phenomenon. *J Epidemiol Community Health.* 2005;59(6):443-449.
