# Supplementary material for: Statin discontinuation and new antipsychotic use after an acute hospital stay vary by hospital
Source: PLoS One. 2020 May 8;15(5):e0232707. doi: 10.1371/journal.pone.0232707 (PMC7209203; doi:10.1371/journal.pone.0232707)
Supplement: S3 Appendix — (DOCX) [file pone.0232707.s003.docx]

**S3 Appendix 3. Post-hoc sensitivity analysis for new antipsychotic use cohort**

A post-hoc sensitivity analysis was conducted to examine if inclusion of a mental health diagnosis as a covariate in our multilevel model (previously described in Appendix 2) affected the odds of new antipsychotic use. This dichotomous mental health diagnosis covariate was created from single level CCS categories 650-663 and 670 (Table 1). Results of the multilevel model with this covariate added are providing in Table 2. The median odds ratio at hospital-level, adjusted for differences in patients remained the same at 1.29 (95% CI: 1.24, 1.34).

| Table 1: Diagnoses included in mental health diagnosis covariate |
| --- |
| Single-level CCS categories |
| 650 Adjustment disorders |
| 651 Anxiety disorders |
| 652 Attention-deficit, conduct, and disruptive behavior disorders |
| 653 Delirium, dementia, and amnestic and other cognitive disorders |
| 654 Developmental disorders |
| 655 Disorders usually diagnosed in infancy, childhood, or adolescence |
| 656 Impulse control disorders, NEC |
| 657 Mood disorders |
| 658 Personality disorders |
| 659 Schizophrenia and other psychotic disorders |
| 660 Alcohol-related disorders |
| 661 Substance-related disorders |
| 662 Suicide and intentional self-inflicted injury |
| 663 Screening and history of mental health and substance abuse codes |
| 670 Miscellaneous mental health disorders |

**Table 2. New antipsychotic use in the year following hospitalization including mental health diagnosis covariate**

|  | OR (95% CI) |
| --- | --- |
| ICU admission, sepsis diagnosis | 1.27 (1.14, 1.42) |
| ICU admission, no sepsis diagnosis | 0.89 (0.85, 0.92) |
| Hospitalization, no ICU | 1.0 |
| Age (unit=5 years) | 0.92 (0.91, 0.92) |
| Race |  |
| White | 1.0 |
| Black | 1.07 (1.01, 1.14) |
| Other | 1.02 (0.97, 1.07) |
| Male | 0.83 (0.79, 0.87) |
| Illness severity (unit=0.01) | 1.02 (1.02, 1.03) |
| Elixhauser-Van Walraven Score | 0.98 (0.98, 0.99) |
| Admission Diagnosis |  |
| Congestive heart failure; non-hypertensive | 0.76 (0.70, 0.82) |
| Nonspecific chest pain | 1.26 (1.16, 1.37) |
| Coronary atherosclerosis and other heart disease | 0.70 (0.63, 0.77) |
| Cardiac dysrhythmias | 0.61 (0.55, 0.67) |
| Alcohol-related disorders | 0.29 (0.28, 0.31) |
| Chronic obstructive pulmonary disease and bronchiectasis | 0.92 (0.84, 1.00) |
| Pneumonia | 0.87 (0.80, 0.95) |
| Skin and subcutaneous tissue infections | 0.89 (0.82, 0.97) |
| Osteoarthritis | 0.37 (0.32, 0.41) |
| Complication of device; implant or graft | 0.77 (0.69, 0.86) |
| Region |  |
| Midwest | 1.00 (0.85, 1.18) |
| Northeast | 1.11 (0.93, 1.34) |
| South | 1.15 (0.99, 1.34) |
| West | 1.0 |
| AHA hospital size |  |
| Large | 0.83 (0.69, 1.00) |
| Medium | 0.97 (0.85, 1.11) |
| Small | 1.0 |
| Hospital complexity |  |
| 1a-Most complex | 0.82 (0.66, 1.03) |
| 1b | 0.70 (0.56, 0.88) |
| 1c | 0.88 (0.72, 1.09) |
| 2 | 0.78 (0.61, 0.98) |
| 3-Least complex | 1.0 |
| Teaching hospital | 1.03 (0.89, 1.19) |
| Admission year |  |
| 2014 | 1.04 (1.01, 1.07) |
| 2015 | 1.04 (1.01, 1.08) |
| 2016 | 1.0 |
| Mental health diagnosis | 12.28 (11.76, 12.82) |

*Note:* For continuous variables, the odds ratios are for a 1 unit change from the mean, except for illness severity and age, which is 0.01 unit change and 5-year change from the mean, respectively.
