# Supplementary figures and images for: Statin discontinuation and new antipsychotic use after an acute hospital stay vary by hospital
Source: PLoS One. 2020 May 8;15(5):e0232707. doi: 10.1371/journal.pone.0232707 (PMC7209203; doi:10.1371/journal.pone.0232707)

**S4 Appendix 4. Cumulative density function of statin fills prior to hospitalization**

**
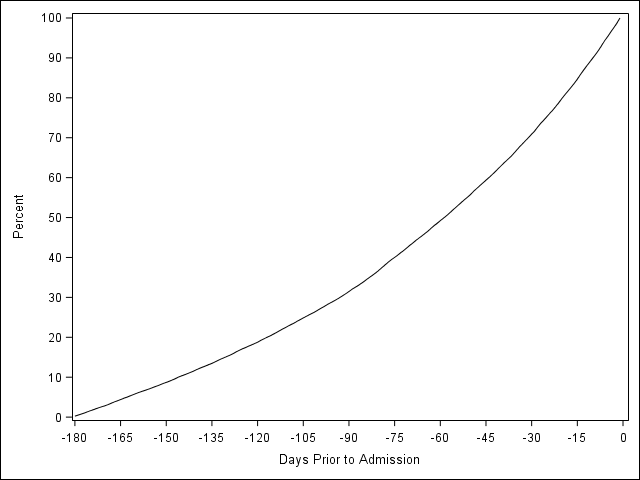
**

Supplement: S4 Appendix — (DOCX) [file pone.0232707.s004.docx]

**S5 Appendix 5. Cumulative density function of antipsychotic fills after hospitalization**

**
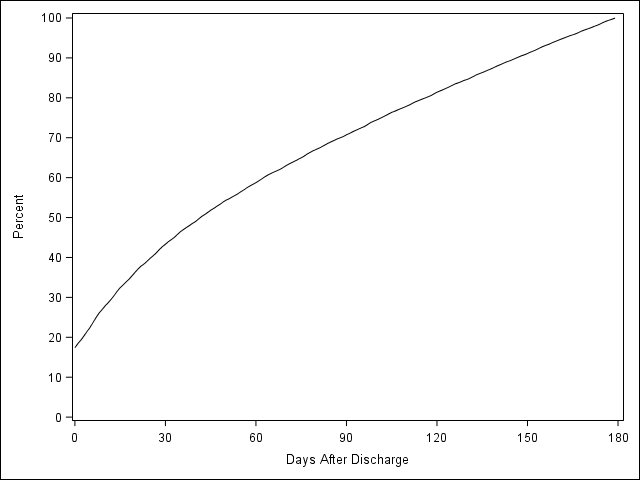
**

Supplement: S5 Appendix — (DOCX) [file pone.0232707.s005.docx]
